# A study protocol for a cluster randomised trial for the prevention of chronic suppurative otitis media in children in Jumla, Nepal

**DOI:** 10.1186/s12901-015-0017-x

**Published:** 2015-09-29

**Authors:** Susan Clarke, Robyn Richmond, Heather Worth, Rajendra Raj Wagle

**Affiliations:** School of Public Health and Community Medicine, University of New South Wales, High St., Kensington, NSW 2305 Australia; Department of Community Medicine and Public Health, Tribhuvan University Institute of Medicine, Maharajganj, Kathmandu, Nepal

**Keywords:** Otitis Media, Hearing, Women’s groups, Community intervention

## Abstract

**Background:**

Chronic Suppurative Otitis Media (CSOM) is the commonest cause of preventable deafness, affecting 164 million people worldwide, 90 % of whom live in low resource countries, such as Nepal. Simple, inexpensive treatment of acute otitis media can prevent the development of CSOM and its sequelae: deafness, abscess, encephalitis, and, rarely, death. CSOM is a disease of poverty and its social determinants: low parental education, overcrowding, poor hygiene and malnutrition. Previous studies have established economic, socio-cultural and geographic barriers to care seeking for childhood illness in the developing world and, in particular, in Nepal. The ultimate aim of this research is to improve the ear health of the children in Jumla, Nepal. The primary outcome is an increase in mother’s knowledge, attitude and practice regarding ear disease in their children. The secondary outcome is a reduction in the prevalence of CSOM in their children.

**Methods/design:**

Using 56 existing women’s self-help groups, sample size, adjusting for clustering and data analysis, is set at 15 groups per arm. A baseline survey of 30 randomly selected groups will be performed, consisting of a knowledge, attitude and practice questionnaire aimed at women who participate in self-help groups, as well as examination of their children’s ears. This will be followed by random allocation, stratified by geography, into 15 intervention and 15 control groups. The intervention groups will participate in three interactive educational sessions at their regular monthly meetings based on *World Health Organisation Primary Ear and Hearing Resource, Basic Level.* The control groups will continue their usual monthly group meetings. At 12 months, a follow-up assessment of both control and intervention groups will be performed, with a repeat women’s survey and repeat ear examination of the children. Data analysis will be by intention to treat and clustering will be considered at every stage. Cluster level data will be analysed using *t*-test and individual level data using mixed effects linear regression and logistic regression random effects model as appropriate.

**Discussion:**

Despite its remote location, Jumla has a vibrant network of health posts and community workers. This project uses existing, local resources and will be undertaken in a way that is consistent with the cultural understanding of the local community in Jumla and acceptable to local care-givers.

**Trial registration:**

Australia and New Zealand Clinical Trials Register, ACTRN12614000231640.

## Background

Chronic Suppurative Otitis Media (CSOM) is a common, preventable cause of deafness, chronic ill-health and, rarely, death in children in low resource countries. CSOM usually arises as a complication of untreated acute otitis media, which is almost universal in young children. Ear infections are so common, that they can seem normal and of low health priority [1, 2]. Acute ear infections are simple and inexpensive to treat, which can prevent CSOM, yet many children receive no assessment or intervention. Simple topical treatment with Ciprofloxacin ear drops has been found to be superior to both oral antibiotics and older combination drops [3–5]. Locally made Ciprofloxacin eardrops are widely and cheaply available in Jumla. CSOM is a disease of poverty and its social determinants. Low parental education level, low parental income, malnutrition, overcrowding, lack of clean water and sanitation are all associated with increased risk of CSOM [1, 6, 7]. The children most at risk of CSOM are also the ones with least access to health education and health care.

The World Health Organisation (WHO) has declared that a prevalence of CSOM greater than 4 % requires emergency measures [1]. At least 50 % of children with CSOM will have significant hearing loss, a global burden of 164 million people, 90 % of whom live in low resource countries, such as Nepal [1]. The most extensive recent prevalence survey in Nepal found that 7.6 % of children aged 5 to 13 years had CSOM [8]. Multiple smaller scale surveys have returned similar results [9–13].

Previous studies have established economic, socio-cultural and geographic barriers to care seeking for childhood illness in the developing world, including Nepal [1, 14–16]. Even in places where appropriate health services are available, they are not fully utilitised [17]. Community based interventions have been trialled to improve engagement with health services and decrease childhood morbidity and mortality [18–20]. The World Health Organisation has produced a primary ear care programme to educate and motivate the community about ear infections and the need to take action to prevent CSOM and hearing loss [21]. This programme is simple and can easily be adapted to cultural contexts and delivered in a socially acceptable setting. There have been few previous successful interventions aimed at prevention and early treatment of CSOM and no published data on the effectiveness of community education or primary ear care training for the community. Consequently, this trial tests the hypothesis that simple ear training can improve maternal practice and reduce the prevalence of the complications of acute ear infections.

## Methods/design

### Study aim

The overarching aim of our study is to improve the ear health of the children in Jumla, Nepal. In order to achieve our aim, our objectives include: improving the knowledge and practice of women regarding the ear health of their children, reducing the prevalence of CSOM and reducing the prevalence of other abnormalities of the tympanic membrane.

### Study design

Our study uses a cluster randomised trial design and is profoundly informed by our previous qualitative study (unpublished), consisting of in-depth interviews with women in Jumla, Nepal. Existing women’s self-help groups will be the unit of randomisation, or cluster. This is a suitable method for an intervention that takes place at the group level and is commonly used in community-based interventions [18, 19, 22–24]. Stratification will be done to avoid sampling bias, as power is better maintained than with matching. The 56 existing groups will be stratified into according to Village Development Committee and the remoteness of their location and distance to the road. Although all of these groups are in a remote location, distance to the road determines accessibility of health and other services. Following stratification, a random sample of 30 groups will be chosen for the study. Randomisation will be done using a computer generated random number sequence in Australia, by a member of the team (HW) not involved in data collection. The selection will then be communicated to the field team leader by internet or telephone. We will seek individual informed consent from each participant woman. After baseline data collection, groups will be randomly allocated to the intervention group and to the control group, stratified by geography. Randomisation will again be performed by a team member in Australia using a computer generated random number sequence and communicated to the field team leader by internet or telephone. The intervention will then be implemented over three consecutive regular monthly meetings of the self-help groups. International Nepal Fellowship is the only non-government organisation working directly with women in the two Village Development Committees, so it is unlikely that any co-interventions might contaminate the study. Women in Jumla are not generally mobile. A small number of women do migrate to India to work with their husbands and these women could be lost to follow-up but the numbers are likely to be very small. In the 2011 census, only 341 women in the entire district of Jumla were absent from their their homes [25]. Follow-up assessment at 12 months, repeating the women’s questionnaire and examination of the children’s ears will complete the study. Ethical approval has been granted by Human Research Ethics Committee, University of New South Wales (Ref# 13361) and Nepal Health Research Council (Ref # 1434).

### Study setting

Our study is set in the isolated, mountain district of Jumla, Nepal. Nepal is a poor country with a Human Development Index 0.54, a rank of 145 out of 187 countries [26]. Jumla has an Human Development Index of 0.35, with a rank of 68 out of 75 districts in Nepal, making it one of the poorest and most disadvantaged districts in an already impoverished nation [27]. There has been little health research done in Jumla, although it was the first region to introduce community based management of childhood pneumonia [28–31]. Most of the population (88.7 %) live in small mountainside villages and eke a bare living from subsistence agriculture [32]. International Nepal Fellowship has agreed to host our research. They have been working in Jumla since 1978, initially offering outpatient and inpatient leprosy and tuberculosis care, later expanding their services to include a Community Development and Rehabilitation team. International Nepal Fellowship has a policy of social inclusion and poverty alleviation. They purposefully include women, low caste people and people living with disabilities in all of their programmes. International Nepal Fellowship has 56 women’s self-help groups, each comprising approximately 20 to 25 women, in two village development committee areas. The groups were formed following extensive community and local government consultation to find villages with the most poor and marginalised people. Consent and participation by villagers eventually led to the formation of the self-help groups. The groups have a distinct geographical catchment within the village and contain neighbours and extended family members. Each group has a monthly meeting facilitated by community workers. At the group meetings members discuss and participate in microfinance, income generation, health education and community mobilisation. Our research is set within these women’s self-help groups.

### Study population

The participants are members of the existing women’s self-help groups and their household children age 12 and under. All members of the study groups will be invited by International Nepal Fellowship staff to participate. In this setting it is likely that all members will agree to participate as the burden on them is very slight. Women who live in a household with a child aged 12 and under will be included in the survey since rural Nepalis mostly live in extended family groups and mothers-in-law are important decision-makers for child health [33–35]. CSOM is a chronic condition and occurs at all ages, with a peak in early childhood. The age of 12 has been chosen as the upper limit as most young children continue to live with their families and attend local schools. Older children are increasingly moving to the district capital for the later years of high school, which would make followup assessment difficult.

### Inclusion criteria

- Women, aged 18 years and over, who are attending International Nepal Fellowship women’s self-help groups in Jumla and who have a child aged 12 years and under living in their household
- The children aged 12 years and under who live in the household of participant women

### Exclusion criteria

**Fig. 1:**
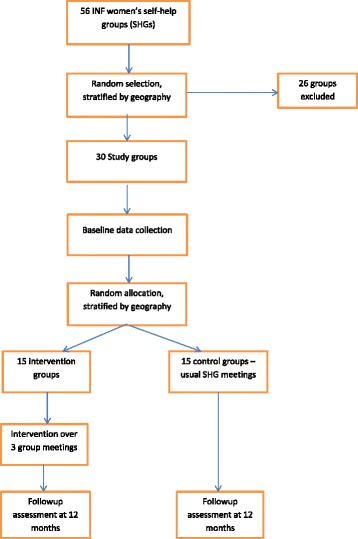
Study design

- Women under the age of 18 years
- Women who do not have a child aged 12 years and under living in their household
- Women who cannot give informed consent because of infirmity or illness
- Children over the age of 12 years
- Participants will not be paid or rewarded individually for their participation (Fig. 1).

### Content of the intervention

The intervention is community-based and participatory and it will not be possible for participants to be blinded after allocation. The design of the intervention is greatly dependent upon the interviews from our exploratory qualitative study. Those interviews with women revealed that they enjoy meeting and learning together in their groups and that they had a strong preference for visual and interactive presentations. Three sessions will take place within the existing regular monthly meeting of the women’s self-help group meeting in the intervention groups. The intervention is based on the WHO *Primary Ear Care Training Programme*, which has been widely adopted in India and other low resource settings. The basic level of the programme aims to educate parents, carers and community health workers about ear infections, their recognition, risk factors, prevention, risk of hearing loss and appropriate care-seeking [21]. Session 1 and Session 2 will be facilitated by the field team leader. Session 3 will be completed by the usual group facilitator. Recap of previous sessions is a normal part of their group meetings. The content of each session is standardised. Attendance at each session will be recorded and a sensitivity analysis performed.

#### Session 1

The first session is an interactive education session using drama and pictures. We have developed a new resource using local photographs based on the *WHO Primary Ear Care Training Programme—Basic Level.* The first of four components shows the importance of the ear and the sense of hearing—demonstrating the usefulness of being able to hear every day noises such as a baby crying, conversation, dogs barking and, for children, hearing in the busy classroom. The second component is identifying signs and symptoms of ear infections including ear pain, discharge, pulling at ears, fever and irritability. The third section details the transmission of ear infections via coughing, sneezing, touching and dirty water. The final section examines ear care, not putting anything in the ear, such as sticks, oil or any or substance, encouraging appropriate care seeking at the local health post and explaining about the medications that will be provided there.

#### Session 2

The second session is a practical session learning hands-on mopping of the ear and correct installation of drops, along with reinforcement of the messages from the first session. This is a very important session as there is little instruction given in the Nepali health system. The diagnosis and treatment is often very appropriate and health posts deliver care at no cost to the patient, but there is frequently a lack of patient engagement, explanation, question answering and demonstration. Therefore, since ear wicking and installation of drops must be performed repeatedly at home to work effectively, it is necessary for care-givers to master the technique.

#### Session 3

The third session is a brief recap of sessions one and two and will include a small laminated card for each woman to take home with pictures of ear-wicking and drop installation in a child’s discharging ear. Each session will be responsive to the cultural context of village life and the extremely difficult daily lives of the women. Participation will be sought at every step and sessions adapted to women’s expressed understanding, questions and needs.

### Outcome measures

#### Primary outcome measure—the questionnaire

The survey instrument for women in the intervention and control groups is a questionnaire. The primary outcome measure is a 25 % difference in knowledge, attitude and practices, regarding ear disease in their children, of women in the intervention group compared to the control group. There are demographic questions such as age, number of children, maternal education, food security, usual health practices followed by questions about knowledge, attitude and practice regarding ear health, hearing, ear disease and health care seeking. The questionnaire uses already validated questions from the Demographic Health Survey (DHS) and Multiple Indicator Cluster Survey (MICS) performed recently in Nepal and is informed by a similar survey conducted in South India [32, 36, 37]. Other questions have been inspired by the *Primary Ear and Hearing Care Training Resource* and the literature on health care engagement [21]. The knowledge section asks the question ‘what do women know about ear infections in their children’, what are the local causes, treatments and complications. The attitude section queries ‘what do women think about ear infections’, is there a low level of ‘threat perception’, are there cultural barriers to seeking care, is there a perception of poor quality care or that ear infections are incurable. Finally, the practice section inquires what they do about them, whom they consult, if anyone, what would help them seek care and whether they know what care is available. The questionnaire has been pre-tested in Australia with a small adjustment made to a single question. The duration in English with educated mothers is 10 mins. Pretesting with village women on the terai (plains) did not reveal any difficulties with the questionnaire. We then undertook a formal pilot study of 20 village women in Jumla, attending INF clinics in Jumla Bazaar, to check for length, understanding and clarity. For these uneducated and illiterate women, the survey was too long and several questions incomprehensible to them. The questionnaire was edited appropriately and five questions removed from the demographic section. The new questionnaire was clear and can be completed in 10-15 mins. The questionnaires will be completed on paper in Nepali by the research assistants trained by the field team leader. Training will take place over two days in Jumla bazaar. A number of interviews will be observed by the field team leader to ensure quality control and all forms will be reviewed regularly to ensure correct completion by assistants. Baseline assessment will be performed prior to allocation into intervention and control groups to avoid bias. Group attendance and financial records are strictly kept at each meeting so it is not possible for an outsider to attend a group meeting unobserved. It is possible that there could be contamination of the control group by an intervention group member offering advice. This is not very likely as extended family mostly live side by side, meaning that grandmothers, aunties and mothers, who might share childcare and advice, are in the same group, or cluster. There are few sources of health information for these mainly illiterate women in this remote region. Followup assessment will be performed 12 months after baseline assessment. The trained research assistants performing followup assessment will be blinded to allocation.

#### Secondary outcome measure—examination of the children’s ears

The other part of the baseline data collection is examination of the children’s ears. The secondary outcome measure is a reduction in the prevalence of CSOM and other abnormalities of the tympanic membrane in the intervention group compared to the control group. We will also offer a health check to all of the children. It is likely that this will be well-received as it is rare for a medical doctor to leave the district capital. The field team leader, an experienced Australian General Practitioner, will examine the children’s ears and take a digital image of their eardrums with a Welch Allyn Digital Macroview Otoscope, if possible, for later verification with an Ear Nose and Throat specialist blinded to allocation. All images will be identified by number only and submitted to the Ear Nose and Throat specialist who will be the final arbiter of diagnosis. CSOM will be diagnosed according to WHO Guidelines with aural discharge of more than two weeks [1]. There is considerable variation internationally about the duration of discharge needed to diagnose CSOM, so children with discharge for longer than six weeks and three months will be documented and analysed separately [38]. Height, weight, middle upper arm circumference, vision, heart and skin checks will also be performed. Weight will be measured to nearest 100 g with Seca 876 scales, height will be measured to nearest 1 mm with Seca 217 mobile height measurement and length to nearest 1 mm with Seca 210 mobile measurement mat. Vision checks will use Revised Sheridan Gardiner Test, which is suitable for illiterate children. Research assistants will be trained in accurate use of equipment and recording of data by the field team leader using WHO Training Course on Child Growth Assessment (2008) [39]. The field team leader will perform skin, heart and ear examinations. Children with medical issues will be referred to health posts or the local hospital, as appropriate. Any child with a current ear infection will also be offered treatment or referral to local health services. There is no specialist ear service in Jumla It would not be ethical to deny treatment to any child. Results of the examination will be recorded on a paper that will also record the ID number of their mother or grandmother**.**

### Harms

Confidentiality will be strictly kept. Completed questionnaires, children’s data sheets and digital images will be identified by number only. The paper forms will be kept in a locked box in the field. Consent forms will be kept in the same box. All paper data will subsequently be stored in the security protected locked filing cabinet in the basement storage of the School of Public Health and Community Medicine, University of New South Wales. Electronic data will be kept on a password protected computer. This research has a very low possibility of causing harm. Women could become distressed while discussing their children’s health. In the unlikely event that followup is needed the field team leader will organise this with local health services. Any children found to have medical conditions will be referred to local health services. The authors are the co-ordinating committee for this research and take responsibility for co-ordination and data management. The endpoint of the research is predetermined and the risk for harm is minimal.

### Sample size calculation

The sample size is calculated on the primary outcome. Using data extracted from questions in the previous qualitative study, sample size for an unclustered study with a 5 % two sided Type 1 error and 80 % power to detect a 25 % difference in mean questionnaire score, would be 114 women in each arm. Both Daly et al. in USA and Srikanth et al. in India found a very low level of knowledge about causes and risk factors for otitis media in mothers of young children [37, 40]. We anticipate a similarly low level in Jumla, and hypothesise that a 25 % increase would be modest and achievable. The cluster sizes are set at the size of the women’s groups which is around 20 women. To adjust for clustering, it is necessary either to establish an approximate intra-cluster correlation coefficient (ICC) or ρ, which measures the similarity of individual responses within clusters, in order to calculate the design effect (the number by which an individual trial must be multiplied to adjust for clustering) or to calculate the between cluster variability, k. These values vary and any estimate can only be approximate. Other studies on neonatal mortality in women’s groups in Nepal have found a very low ICC of 0.00644 [24]. Tielsch et al. [41] calculated a design effect of 1.23 with their study of supplements to prevent malnutrition in children. Mullany et al. [22] used a design effect of 2.0 for their study looking for a 25 % reduction in omphalitis in newborns in rural Nepal. Slightly further afield in Bangladesh, Aboud et al. [23] calculated an ICC of 0.03 for a responsive feeding and stimulation intervention to clusters of rural women and children. Pagel et al. [42] reviewed ICC for interventions around perinatal outcomes in low resource countries and found universally low ICC for maternal and neonatal mortality but higher ICC for other interventions such as skilled birth attendance. For example, in India and Bangladesh skilled birth attendance ranged from ICC 0.02-0.04. The Design Effect (DEff) is the number by which the sample size for an individual randomised controlled trial must be multiplied to give the equivalent power to a cluster randomised trial. The Design Effect also considers the number of individuals in each cluster *– m.* Using the equation DEff = 1 + (*m*-1) ρ, assuming ρ = 0.03, would give a DEff = 1.6, or 182 women per arm. Using the safe equation DEff = 1 + (m-1) ρ, assuming ρ = 0.05, would give a DEff = 1.95, or 223 women per arm. This would translate into 11 clusters per arm. To account for stratifying, we have added a conservative extra two clusters per arm, which would make 13 clusters per arm. To enable robust cluster analysis a minimum of 15 clusters is ideal. This will provide a generous total sample of approximately 600 women in 30 clusters, 15 in the intervention and 15 in the control arm.

### Statistical analysis

Analysis will be by intention-to-treat, all study participants will be included in the analysis, even if lost to follow-up. Analysis will be adjusted for clustering. Every effort will be made to have complete data but missing data will not be ignored in the analysis. Data will be checked and cleaned before being entered into Statistical Package for the Social Sciences. At baseline a comparison profile of both intervention and control groups will be provided. The primary and secondary outcomes - difference in mean questionnaire score and prevalence of CSOM - will be compared between the two groups. Data will be analysed at both individual and cluster level. Cluster level analysis is very robust and, with 15 clusters in each arm, individual level analysis, adjusted for clustering ought also to be very robust. For the primary outcome, a summary measure, the mean score of the questionnaire, will be calculated and then the intervention and control group compared for significance with a *t*-test at the cluster level. For individual level analysis, we plan to use mixed effects linear regression, which adjusts for the effect of clustering. The secondary outcome is a proportion, so we plan to analyse individual level data using logistic regression random effects model, using quadrature checks, which also adjusts for clustering. Since the age range is quite broad we will consider each standard age grouping in our analysis: under one year, under five years, and five years and over. We will also analyse duration of ear discharge as this is strongly correlated with prognosis. Covariates including socioeconomic status, education, caste, geography and number of household members will be considered.

## Discussion

This trial will be the first randomised controlled trial to try to establish the effectiveness of a community based intervention to improve the ear health of children in Nepal. We have designed an intervention that is consistent with community values, expectations and experiences, using local existing resources. We have postulated that asking women what they want to know, how they like to learn and what is important to them, formulating an intervention on those principles, and then delivering it in a familiar, acceptable setting will increase its uptake. We will direct women to seek help at existing health services – health posts and outpatient clinics. Health posts offer treatment and medication at no cost to the patient and, in Jumla, are readily accessible by foot. However, availability of care is not equivalent to access to care. Lack of knowledge, lack of threat perception, disempowerment, gender inequity all conspire together with poverty and geographical isolation to prevent adequate treatment of ear disease in children. Women’s groups have been a potent means of health education, empowerment and training in Nepal, improving maternal and child health. They are the ideal medium for a participatory, culturally contextual, engaging intervention to attempt to prevent CSOM.

## Trial status

Commenced data collection.

## Abbreviations

CSOM: Chronic suppurative otitis media
DEff: Design effect
ICC: Intracluster correlation coefficient
WHO: World Health Organisation

## References

[1] World Health Organisation (2004). Chronic suppurative otitis media: burden of illness and management options.

[2] Monasta L, Ronfani L, Marchetti F, Montico M, Brumatti LV, Bavcar A (2012). Burden of disease caused by otitis media: systematic review and global estimates. PLoS One.

[3] Leach A, Wood Y, Gadil E, Stubbs E, Morris P (2008). Topical ciprofloxin versus topical framycetin-gramicidin-dexamethasone in Australian aboriginal children with recently treated chronic suppurative otitis media: a randomized controlled trial. Pediatr Infect Dis J.

[4] Macfadyen C, Acuin J, Gamble C. Systemic antibiotics versus topical treatments for chronically discharging ears with underlying eardrum perforations. Cochrane Database Syst Rev. 2006, Issue 1.Art.No.:CD005608. doi:10.1002/14651858.CD005608. 10.1002/14651858.CD005608 16437533

[5] van der Veen EL, Rovers MM, Albers FW, Sanders EA, Schilder AG (2007). Effectiveness of trimethoprim/sulfamethoxazole for children with chronic active otitis media: a randomized, placebo-controlled trial. Pediatrics.

[6] Shaheen MM, Raquib A, Ahmad SM (2012). Prevalence and associated socio-demographic factors of chronic suppurative otitis media among rural primary school children of Bangladesh. Int J Pediatr Otorhinolaryngol.

[7] Taipale A, Pelkonen T, Taipale M, Bernardino L, Peltola H, Pitkäranta A (2011). Chronic suppurative otitis media in children of Luanda, Angola. Acta Paediatr.

[8] Adhikari P (2009). Pattern of ear diseases in rural school children: experiences of free health camps in Nepal. Int J Pediatr Otorhinolaryngol.

[9] Devkota B, Baidya P, Chettri H (2003). Health needs assessment of the primary school children in Nepal. J Nepal Health Res Counc.

[10] Little P, Bridges A, Guragain R, Friedman D, Prasad R, Weir N (1993). Hearing impairment and ear pathology in Nepal. J Laryngol Otol.

[11] Adhikari P, Kharel B, Ma J, Baral DR, Pandey T, Rijal R (2008). Pattern of otological diseases in school going children of Kathmandu valley. Arq Int Otorrinolaringol.

[12] Rijal A, Joshi R, Regmi S, Malla N, Dhungana A, Jha A (2011). Ear diseases in children presenting at Nepal Medical College Teaching Hospital. Nepal Medical College journal: NMCJ.

[13] Sigdel B, Nepali R. Pattern of Ear Diseases among Paediatric ENT Patient: An Experience from Tertiary Care Centre, Pokhara, Nepal. Journal of Nepal Paediatric Society. 2012;32(2):142-145.

[14] Najnin N, Bennett CM, Luby SP (2011). Inequalities in care-seeking for febrile illness of under-five children in urban Dhaka, Dangladesh. J Health Popul Nutr.

[15] LeVine RA, LeVine SE, Rowe ML, Schnell-Anzola B (2004). Maternal literacy and health behavior: a Nepalese case study. Soc Sci Med.

[16] Pokhrel S (2007). Determinants of parental reports of children’s illnesses: empirical evidence from Nepal. Soc Sci Med.

[17] Ergler CR, Sakdapolrak P, Bohle H-G, Kearns RA (2011). Entitlements to health care: Why is there a preference for private facilities among poorer residents of Chennai, India?. Soc Sci Med.

[18] Bhandari N, Bahl R, Mazumdar S, Martines J, Black RE, Bhan MK (2003). Effect of community-based promotion of exclusive breastfeeding on diarrhoeal illness and growth: a cluster randomised controlled trial. Lancet.

[19] Penny ME, Creed-Kanashiro HM, Robert RC, Narro MR, Caulfield LE, Black RE (2005). Effectiveness of an educational intervention delivered through the health services to improve nutrition in young children: a cluster-randomised controlled trial. Lancet.

[20] Rubenstein R, Lane SD (2012). From intervention to outcome: the relationship between knowledge and behavior in a trachoma control project. J Healthcare, Science and the Humanities.

[21] World Health Organization (2006). Primary ear and hearing care training resource.

[22] Mullany LC, Darmstadt GL, Khatry SK, Katz J, LeClerq SC, Shrestha S (2006). Topical applications of chlorhexidine to the umbilical cord for prevention of omphalitis and neonatal mortality in southern Nepal: a community-based, cluster-randomised trial. Lancet.

[23] Aboud FE, Akhter S (2011). A cluster-randomized evaluation of a responsive stimulation and feeding intervention in Bangladesh. Pediatrics.

[24] Manandhar DS, Osrin D, Shrestha BP, Mesko N, Morrison J, Tumbahangphe KM (2004). Effect of a participatory intervention with women’s groups on birth outcomes in Nepal: cluster-randomised controlled trial. Lancet.

[25] Government of Nepal National Planning Commission Secretariat and Central Bureau of Statistics (2012). National Population and Housing Census 2011.

[26] United Nations Development Programme. International Human Development Indicators 2014. [http://hdr.undp.org/en/countries/profile/NPL]

[27] United Nations Field Co-ordination Office (UNFCO) Nepalgunj Nepal. An Overview of the Mid-Western Region of Nepal. [www.un.org.np/sites/default/files/jumla_district_profile.pdf]

[28] Pandey MR, Daulaire NMP, Starbuck ES, Houston RM, McPherson K (1991). Reduction in total under-five mortality in western Nepal through community-based antimicrobial treatment of pneumonia. Lancet.

[29] Dawson P, Pradhan Y, Houston R, Karki S, Poudel D, Hodgins S (2008). From research to national expansion: 20 years’ experience of community-based management of childhood pneumonia in Nepal. Bull World Health Organ.

[30] Thapa N, Chongsuvivatwong V, Geater AF, Ulstein M, Bechtel GA (2000). Infant death rates and animal-shed delivery in remote rural areas of Nepal. Soc Sci Med.

[31] Bishai D, Niessen LW, Shrestha M (2002). Local governance and community financing of primary care: evidence from Nepal. Health Policy Plan.

[32] Ministry of Health and Population (MOHP) [Nepal]; New ERA and ICF International Inc. Nepal Demographic and Health Survey 2011. Kathmandu, Nepal: Ministry of Health and Population, New ERA and ICF International, Calverton, Maryland; 2012. [dhsprogram.com/publications/publication-fr257-dhs-final-reports.cfm]

[33] Ji Y (2013). Negotiating marriage and schooling: Nepalese women’s transition to adulthood. Ann Am Acad Polit Soc Sci.

[34] Link CP (2010). Husbands, wives and in-laws: family dynamics and child-bearing behaviour in Nepal.

[35] Masvie H (2006). The role of Tamang mothers-in-law in promoting breast feeding in Makwanpur District, Nepal. Midwifery.

[36] Central Bureau of Statistics and UNICEF Nepal (2012). Nepal Multiple Indicator Cluster Survey 2010. Mid and Far Western Regions, Final Report.

[37] Srikanth S, Isaac R, Rebekah G, Rupa V (2009). Knowledge, attitudes and practices with respect to risk factors for otitis media in a rural South Indian community. Int J Pediatr Otorhinolaryngol.

[38] Morris PS, Leach AJ, Silberberg P, Mellon G, Wilson C, Hamilton E (2005). Otitis media in young Aboriginal children from remote communities in Northern and Central Australia: a cross-sectional survey. BMC Pediatr.

[39] World Health Organisation (2008). Training course on child growth assessment.

[40] Daly KA, Selvius RE, Lindgren B (1997). Knowledge and attitudes about otitis media risk: implications for prevention. Pediatrics.

[41] Tielsch JM, Khatry SK, Stoltzfus RJ, Katz J, LeClerq SC, Adhikari R (2006). Effect of routine prophylactic supplementation with iron and folic acid on preschool child mortality in southern Nepal: community-based, cluster-randomised, placebo-controlled trial. Lancet.

[42] Pagel C, Prost A, Lewycka S, Das S, Colbourn T, Mahapatra R (2011). Intracluster correlation coefficients and coefficients of variation for perinatal outcomes from five cluster-randomised controlled trials in low and middle-income countries: results and methodological implications. Trials.

